# New Perspectives on Long COVID Syndrome: The Development of Unusually Delayed and Recurring Pericarditis After a Primary SARS-CoV-2 Infection

**DOI:** 10.7759/cureus.25559

**Published:** 2022-06-01

**Authors:** Sujoy Khasnavis, Michael Habib, Fawzi Kaawar, Stacy Lee, Aida Capo, Adam Atoot

**Affiliations:** 1 Internal Medicine, St. George's University School of Medicine, True Blue, GRD; 2 Internal Medicine, Hackensack University Medical Center, North Bergen, USA; 3 Anesthesiology, Hackensack University Medical Center, North Bergen, USA; 4 Pulmonary and Critical Care Medicine, Hackensack University Medical Center, North Bergen, USA; 5 Internal Medicine, Palisades Medical Center, New Jersey, USA

**Keywords:** elevated d-dimer, pericardial window, effusion, cardiac, inflammatory, extrapulmonary, pandemic, pericarditis, delayed manifestations, covid-19

## Abstract

Since the start of the COVID-19 pandemic in early 2020, pericarditis has been identified as a COVID-19 complication. We report a case where the development of pericarditis was unusually delayed after the initial COVID infection. The reported onset of pericarditis and pericardial effusion is anywhere from a few days to a few months after infection. Our case surmises that a latent complication of COVID-19 can manifest more than one year after the initial infection.

A forty-three-year-old male with a past medical history of SARS-CoV-2 infection in September 2020 presented in September 2021 and January 2022 with recurrent sharp chest pain and shortness of breath. During both admissions, he was diagnosed with acute pericarditis, and his workup was significant for elevations in D-dimer and CRP as well as pericardial and pleural effusions.

Recurring pericardial symptoms and persistent elevations in D-dimer and CRP point toward a COVID etiology, particularly in the absence of other factors associated with pericarditis. Our case highlights the importance of recognizing this latent complication one year after the initial infection and how the symptoms can persist beyond the one-year period.

## Introduction

SARS-CoV-2 has been frequently implicated in respiratory diseases after the WHO declared the SARS-CoV-2 pandemic in early 2020. As the novel virus has been studied further over the past two years, it has been linked to diseases of other organ systems as well [1]. We now know and are continuing to discover the extrapulmonary manifestations of COVID, some of which are neurological, psychiatric, cardiovascular, gastrointestinal, renal, and integumentary in nature [1]. Among the cardiovascular manifestations of COVID includes pericardial disease and reports have described various pericardial symptoms associated with the virus [1-5]. Some cases have described the manifestation of classical features of pericarditis and tamponade requiring evacuation of fluid from the pericardial sac [2-4]. Others have described features of pericarditis and pericardial effusion that required pharmacological therapy only [5].

Kermani et al. have identified four mechanisms by which pericardial disease can develop. In the direct pathway, SARS-CoV-2 binds myocardial angiotensin converting enzyme-2 (ACE2) receptors and activates a signal pathway that leads to myocardial injury and cardiomyopathy, and ultimately to a pericardial effusion [1]. The virus can also colonize the pericardial fluid itself and lead to inflammation of the pericardium that way [1]. They also describe indirect pathways where SARS-CoV-2 induces cytokine storms and oxidative stress. Macrophages and endothelial cells are activated during this storm, which subsequently leads to the release of TNF-α, IL-1, IL-6, and IL-8, followed by pericardial inflammation and effusion [1]. Lastly, COVID-induced acute respiratory distress syndrome (ARDS) and hypoxia can lead to pulmonary hypertension and myocardial injury, followed by left ventricular ejection dysfunction and pericardial effusion [1]. Treatment of COVID-induced pericardial disease depends on effusion size and hemodynamic stability [1]. Patients with a small to moderate effusion and hemodynamic stability can be treated with two weeks of non-steroidal anti-inflammatory drugs (NSAIDs) and three months of colchicine as well as oral steroids where severe respiratory disease is observed or where NSAIDS have failed [1]. Patients with large effusions or hemodynamic instability are considered for pericardiocentesis if coagulopathy is not a concern and surgical drainage if coagulopathy is a concern [1].

## Case presentation

**Figure 1:**
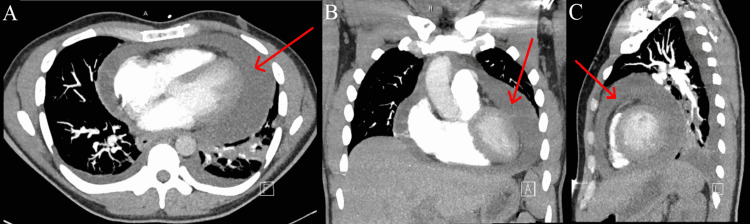
(A) CT PE protocol transverse section of thorax on September 2021. Red arrow marks large pericardial effusion. (B) CT PE protocol coronal section of thorax on September 2021. Red arrow marks large pericardial effusion. (C) CT PE protocol sagittal section of thorax on September 2021. Red arrow marks large pericardial effusion.

**Figure 2:**
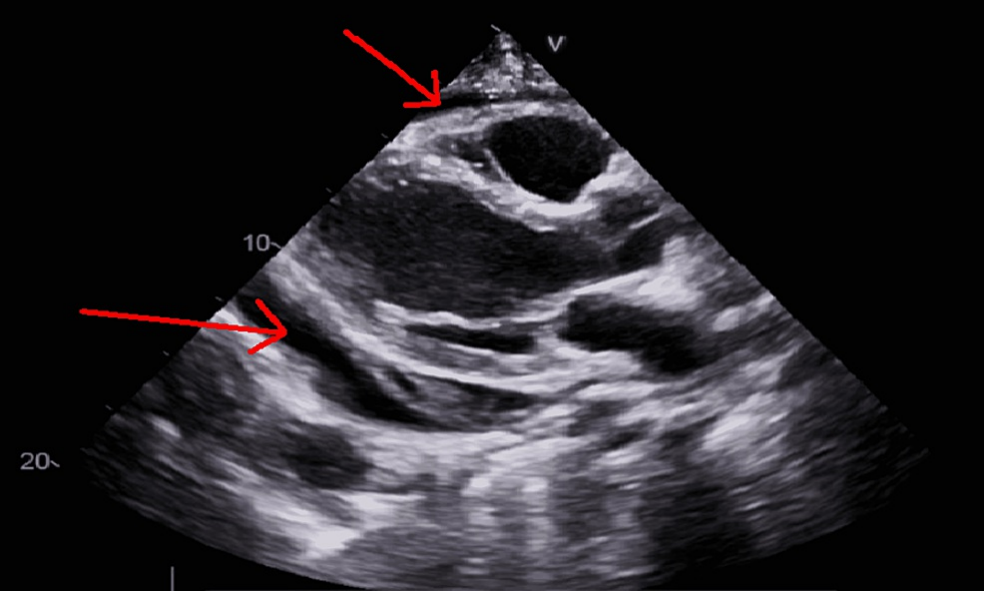
Transthoracic echocardiogram on September 2021. Red arrows point to large pericardial effusion.

**Figure 3:**
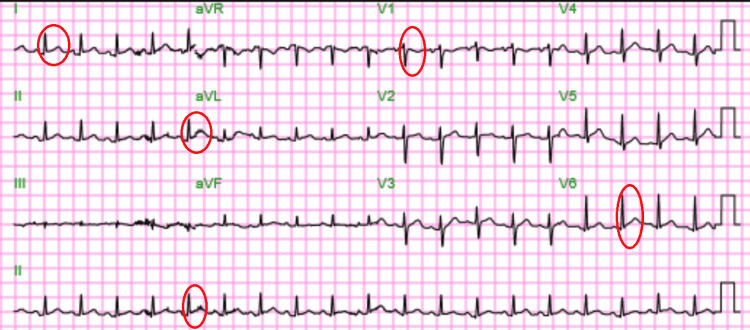
12 lead EKG on September 2021. Sinus tachycardia with diffuse ST elevations suggestive of acute pericarditis. Red circles point out ST elevations in leads I, II, aVL, V1, and V6.

**Figure 4:**
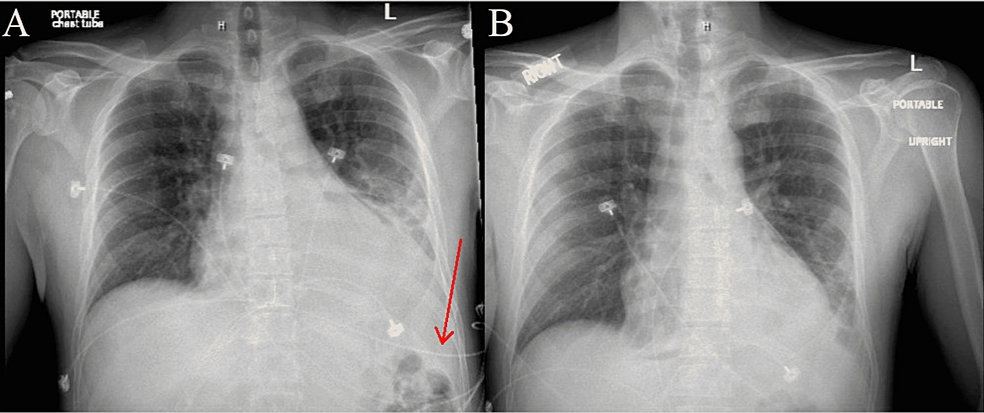
(A) AP chest X-ray on September 2021 prior to pericardial window. Cardiomegaly is present and red arrow points to small left pleural effusion. (B) AP chest X-ray on September 2021 after pericardial window. Cardiomegaly is improved and size of cardiac silhouette is reduced.

A forty-three-year-old male with a past medical history of SARS-CoV-2 infection presented with a six-day history of severe pleuritic chest pain and shortness of breath in September 2021. The pain was worse when lying flat, causing him to cough. It was described as a stabbing pain, located in the mid-epigastric and left side of his chest, rated 10/10 in severity. There were no alleviating factors. The patient denied having any recent fevers, chills, itching of the eyes, runny nose, sore throat, headaches, numbness, dizziness, nausea, vomiting, abdominal pain, myalgias, weakness, or other symptoms prior to this episode of chest pain. He had no known allergies and was not on any medications. The patient had no significant surgical or medical history with the exception of the SARS-CoV-2 infection, which he had experienced one year prior in September 2020. The patient's wife had tested positive for SARS-CoV-2 at that time, and soon thereafter, the patient began to experience COVID-like symptoms as well. He went to an urgent care center where he was confirmed positive for SARS-CoV-2 by a nasopharyngeal swab test and then went home to quarantine for the remainder of the infection period. His vaccinations were up to date except for COVID, for which he was not vaccinated at all. He consumed a regular diet and denied the use of alcohol, cigarettes, and illicit substances. He had no travel history, no exposure to workplace hazards, and no history of sexually transmitted diseases. While the family history was significant for lupus and rheumatoid arthritis in his mother, the patient himself had no history of autoimmune disorders. Upon admission blood pressure was 127/88 mm Hg, heart rate was 87/min, respirations were 18/min, temperature was 98.2 °F, and SpO2 was 99% on room air. On the physical exam, there was jugular venous distention and muffled heart sounds. There was reproducible tenderness to palpation in the mid-epigastric region. All other examination findings were normal. A viral PCR nasopharyngeal swab test was ordered to determine if the patient was experiencing another SARS-CoV-2 infection and the test came back negative. The full blood count, urea and electrolytes, coagulation profile, and liver function tests were normal. The hepatitis panel, virology serology panel, and HIV test were all negative. An autoimmune profile was ordered, which consisted of antinuclear antibody, rheumatoid factor, anti-smooth muscle antibody, dsDNA, C3, C4, and total IgG, all of which were negative. However, elevated values were seen in CRP and D-dimer. The CRP level was 191.8 mg/L and the D-dimer was 8,517 ng/mL. The CT PE protocol was negative for PE, but a large pericardial effusion was noted (Figure 1A-1C). An echocardiogram also showed excess fluid in the pericardial sac (Figure 2). An EKG showed ST-segment elevation in most leads with PR interval depression, suggesting pericarditis (Figure 3). An initial chest X-ray prior to the pericardial window showed clear lungs with an enlarged cardiac silhouette and a left-sided pleural effusion (Figure 4A). The patient underwent a pericardial window with drainage of 500 mL of fluid, and a chest tube was placed to drain residual fluid. The final chest X-ray after the pericardial window showed improvement in cardiomegaly (Figure 4B). After the procedure, the patient was transferred to the ICU, and treatment with naproxen 500 mg BID and colchicine 0.6 mg BID was initiated. Biopsy of the pericardium showed acute and chronic moderate fibrinous inflammation with dense fibrosis. Pericardial fluid analysis with ThinPrep and cell block showed no malignancy, but numerous acute and chronic inflammatory cells were present. The pericardial tube was removed two days later, and the patient-reported improvement in pain. The patient was discharged six days later on colchicine 0.6 mg BID for three months, naproxen 500 mg BID, and protonix 40 mg daily for two weeks and advised to follow up with the primary care physician (PCP) within a week.

**Figure 5:**
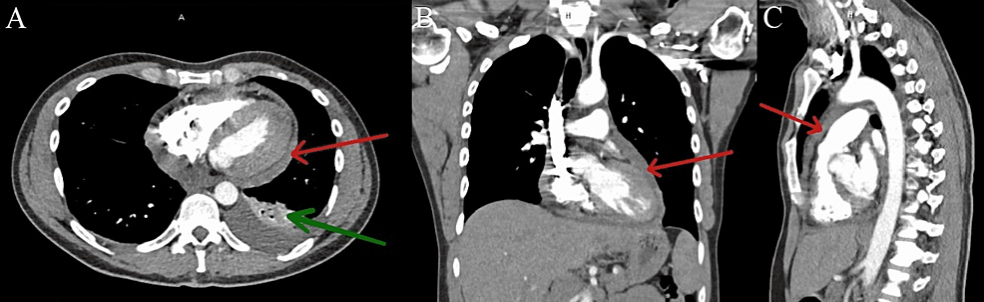
(A) CT PE protocol transverse view of thorax on January 2022. Red arrow shows small pericardial effusion and green arrow shows small left pleural effusion. (B) CT PE protocol coronal view of thorax on January 2022. Red arrow shows small pericardial effusion. (C) CT PE protocol sagittal view of thorax on January 2022. Red arrow shows small pericardial effusion.

**Figure 6:**
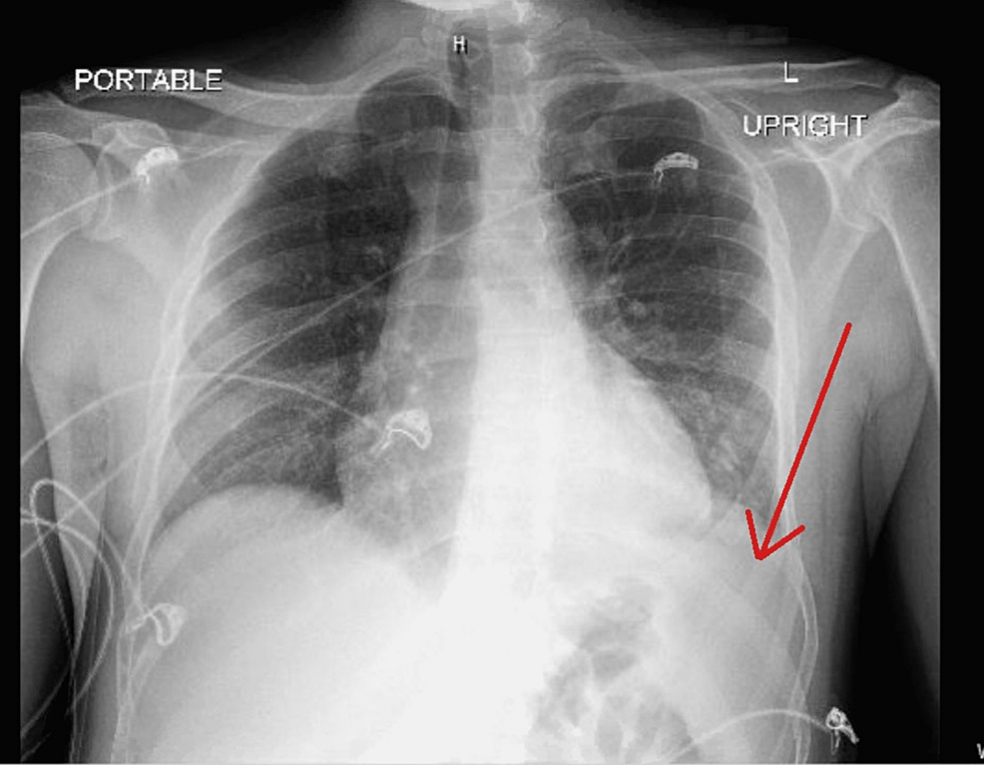
AP chest X-ray on January 2022. Red arrow points to small left pleural effusion.

**Figure 7:**
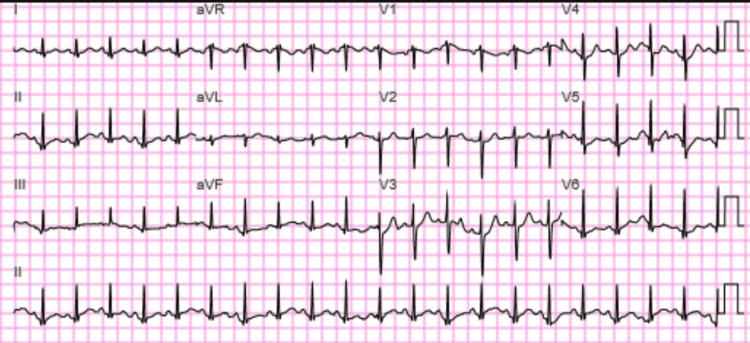
12 lead EKG on January 2022. Sinus tachycardia and widespread nonspecific T wave abnormalities.

The patient presented with another episode of chest pain in January 2022. The pain had started one week prior to presentation and had been worsening since the onset. He described the pain as sharp, located in the substernal area, radiating bilaterally, constant, and 7/10 in intensity. There were no alleviating factors, but it worsened with lying supine and deep inspiration. He also reported shortness of breath and palpitation. On review of systems, the patient again denied any fevers, chills, eye itching, runny nose, sore throat, headaches, numbness, dizziness, nausea, vomiting, abdominal pain, myalgias, weakness, or other symptoms prior to the onset of chest pain. He also denied exposure to alcohol, cigarettes, illicit substances, recent travel, workplace hazards, and sexually transmitted diseases. He was hemodynamically stable while labs again revealed a D-dimer elevation of 7000 ng/mL. The CT PE protocol did not reveal any PE but did reveal a small pericardial effusion (Figure 5A-5C). A chest X-ray revealed a small left pleural effusion and a trace right pleural effusion (Figure 6). His EKG showed sinus tachycardia and widespread ST depressions (Figure 7). He received treatment with lorazepam, ketorolac tromethamine, 1 L of normal saline bolus, and morphine. He was then admitted with the diagnosis of acute pericarditis for further management. He was maintained on NSAIDs and colchicine during the hospital stay. Two days later, he was discharged and instructed to continue taking NSAIDS and colchicine for another two weeks.

## Discussion

The time gap between COVID infection and pericardial disease, along with the pathogenesis of the pericardial disease, is quite variable. Heidari et al. report on a 28-year-old male with no previous illnesses who began experiencing COVID symptoms about one week before presenting to the hospital for chest pain and a diagnosis of hemorrhagic tamponade [4]. They reported isolating bloody fluid on pericardiocentesis with a glucose level of 68 mg/dl, RBC of 320,000, WBC of 13000 with 70% polymorphonuclear cells and 30% mononuclear cells, protein of 4.9 g/dl, LDH of 233 u/l, and negative adenosine deaminase [4]. They report that pericardial fluid testing for SARS-CoV-2 was unavailable but nasopharyngeal swab tests were done twice and came back positive each time, leaving COVID as the only possible etiological factor for tamponade [4]. They go on to report that this delayed pericarditis could have been due to either the primary infection or secondary infection and that confirmation was not possible due to the lack of interim PCR testing [4]. Soewono et al. report on a 30-year-old man presenting with sharp and severe pleuritic chest pain six weeks after testing positive for symptomatic COVID-19 [5]. They reported that the patient was COVID positive by PCR with COVID antibodies on admission but had no other health concerns [5]. They postulate that the cause of his pericarditis was likely a delayed effect of cytokines such as interleukin [IL]-1β, IL-6, IL-8, IL-2, and tumor necrosis factor-α with elevations in D-dimer and C-reactive protein as well [5]. Farina et al. report on a 59-year-old male who was diagnosed with symptomatic COVID by nasopharyngeal swab and developed pericardial effusion with right heart collapse 23 days later [6]. They performed a cytological analysis of the pericardial fluid at the 23-day mark, which yielded erythrocytes, mononucleate elements, lymphocytes, and positive SARS-CoV-2 based on rRT-PCR testing [6]. They also repeated blood and nasopharyngeal tests at this time, which came back negative, suggestive of viral persistence in the pericardial fluid [6]. Kaminski et al. report on a 65-year-old male who had a symptomatic COVID infection with full recovery and developed pericarditis 70 days afterward with a positive COVID test in the pericardial fluid [7].

In our case, the manifestation of pericardial symptoms over one year after the initial infection was a very striking development. Although long COVID symptoms have been identified in other cases, the time gap in our case is an outlier, with symptoms developing between 1 and 1.5 years after the primary infection. Moreover, the patient’s D-dimer and CRP levels were elevated long after the initial infection, which is consistent with the long-term D-dimer and CRP elevations reported in the cases of Kaminski et al. and Soewono et al. These long-term elevations, along with the recurring pericardial symptoms as well as the absence of other etiological factors, point toward SARS-CoV-2 as the likely contributor to our patient’s pericardial disease. The pathogenesis of the patient’s condition requires further study. Acute and chronic inflammation and fibrosis were noted in the patient’s pericardial analyses, but no determination could be made on whether an active infection of the pericardium was involved. Further investigations would definitively provide an answer on whether the delayed manifestations are due to a pure inflammatory process, direct COVID-19 toxicity, or a combination of COVID and other factors.

## Conclusions

Latent complications of SARS-CoV-2 vary as to when they will manifest and are frequently reported days to months after initial infection. However, it is worth noting that manifestations can be delayed much longer, for up to one year and even further, beyond the primary infection. This case highlights the need to consider SARS-CoV-2 as a source of morbidity long after a patient has recovered from the initial infection. This is especially crucial since SARS-CoV-2 contributes to long-standing D-dimer and CRP elevations, recurrent symptoms, and poorer long-term health. Moreover, the unusual pathogenesis in this case points toward an unknown mechanism of disease development and warrants further study to determine how delayed manifestations may differ in mechanism from earlier onset manifestations. This will also unravel if delayed manifestations are due to the primary SARS-CoV-2 infection, pure inflammation, or a combination of SARS-CoV-2 and other factors.
